# Association of Granulocyte Colony-Stimulating Factor Treatment with Risk of Brain Metastasis in Advanced Stage Breast Cancer

**DOI:** 10.3390/ijms251910756

**Published:** 2024-10-06

**Authors:** Yun-Sheng Tai, John Hang Leung, Shyh-Yau Wang, Henry W. C. Leung, Agnes L. F. Chan

**Affiliations:** 1 Department of Breast Surgery, An-Nan Hospital, China Medical University, Tainan 709, Taiwan; 073889@tool.caaumed.org.tw; 2 Department of Obstetrics and Gynecology, Ditmanson Medical Foundation Chia-Yi Christian Hospital, Chia-Yi 600, Taiwan; pc1598jl@gmail.com; 3 Department of Radiology, An-Nan Hospital, China Medical University, Tainan 709, Taiwan; 023930@tool.caaumed.org.tw; 4 Department of Radiation Oncology, An-Nan Hospital, China Medical University, Tainan 709, Taiwan; 5 Department of Pharmacy, Kaohsiung Show Chwan Memorial Hospital, Kaohsiung 821, Taiwan

**Keywords:** GCSF, neutropenia, brain metastasis, stage IV breast cancer

## Abstract

The routine use of granulocyte colony-stimulating factor (GCSF) is not recommended for the prevention or treatment of chemotherapy-induced neutropenia or febrile neutropenia because risks associated with certain types of cancers, distant organ metastases, and primary tumor growth cannot be excluded. We examined the association between GCSF use and the incidence of brain metastasis (BM), as well as BM-free survival (BMFS). This retrospective cohort study included 121 stage IV breast cancer patients without confirmed BM at the time of diagnosis and who received at least one course of systematic chemotherapy or target therapy at a tertiary teaching hospital between 1 January 2014 and 31 December 2022. The effect of GCSF use on BM was assessed with other confounding factors in Cox regression analyses. In this retrospective cohort, patients who received GCSF treatment had a significantly higher incidence of BM than those who did not (34.9% vs. 13.8%, *p* = 0.011). Univariate Cox regression analysis showed that GCSF use, menopause status, hormone treatment, HER2 treatment, cumulative dosage, dosage density, and neutropenia were independent risk factors for BMFS (*p* < 0.05). GCSF users had a higher risk of BM (adjusted HR: 2.538; 95% CI: 1.127–5.716, *p* = 0.025) than nonusers. BM risk was significantly associated with those with neutropenia (RR: 1.84, 95% CI: 1.21, 2.80) but not with those without neutropenia (RR: 0.59, 95% CI: 0.41–0.84, Interaction *p*-value *<* 0.05). The higher the dose density of GCSF, the higher the risk compared with those who do not use GCSF (*p* for trend < 0.01). These preliminary results suggest that GCSF is associated with BM in patients with stage IV breast cancer who did not have BM at initial diagnosis. Further comprehensively designed large-scale observational studies are needed to confirm our preliminary results.

## 1. Introduction

Breast cancer is both the second and fourth leading cause of cancer death among women in the United States and Taiwan, respectively. One-third of women diagnosed with metastatic breast cancer in the United States survive at least 5 years, and some may survive 10 or more years after diagnosis [1,2,3]. If the tumor metastasizes to distant parts of the body, the number of patients who can survive for 5 years is about 30% in the United States and 25.7% in Taiwan [4,5]. The standard treatment for localized breast cancer with neoadjuvant or adjuvant chemotherapy is an anthracycline- and taxane-based regimen combined with surgery and radiation therapy [6]. The main cause of increased breast cancer mortality and treatment failure is late-stage tumor metastasis [7]. Approximately 10% to 15% of patients with stage IV breast cancer develop brain metastases. In particular, BM is more likely to occur in patients with the more aggressive HR−/HER2− subtypes of breast cancer (TNBC 11%), HR−/HER2+ (4%) [8,9]. According to the 2017–2021 US SEER 22 database study, the percent of female breast cases by cancer subtype were HR+/HER2− (70%) and HR+/HER2+ (10%) [9]. Breast cancer can also metastasize to the bones, lungs, or liver, and only in 17% of patients with brain metastases did they spread to the brain [8]. Therefore, understanding the risk factors for distant metastasis may help to select novel and effective treatment options to reduce the risk of metastasis, improve cure rates, and reduce patients’ cancer suffering.

Recombinant human granulocyte colony-stimulating factor (GCSF) is commonly used in cancer patients after myelosuppressive cytotoxic chemotherapy. It reduces the potential risk of infection and hospitalization due to neutropenia, increases the intensity of chemotherapy, and improves overall survival (OS) [10]. It is also a major regulator of neutrophil generation and differentiation, inducing the proliferation and differentiation of myeloid progenitor cells [11]. In a recently published review, accumulating evidence suggests that GCSF is present in the tumor microenvironment and promotes malignant tumor progression and metastasis, leading to poor prognosis and reduced patient survival [12,13]. GCSF may be produced in a variety of cancers, including colon, breast, thyroid, pancreatic, bladder, lung, liver, glioma, and brain metastasis [13,14,15,16,17,18,19,20,21]. A recently published special report by Kono et al. showed that the effect of GCSF produced by tumors can be reflected in the imaging findings of some tumors, such as breast cancer, lung cancer, liver cancer, pancreatic cancer, and colon cancer [22].

The mechanism underlying the effect of GCSF may involve the activation of three major signaling pathways (JAK/STAT, PI3K/AKT, and MAPK/ERK) or the modulation of inflammation and the presence of immunomodulatory effects by mediating innate and adaptive immune responses [12]. The mechanism by which tumor-derived GCSF significantly increases the metastasis-promoting activity of neutrophils via the PI3K-AKT and NF-κB pathways and activates the GCSF-RLN2-MMP-9 axis was recently reported in an analysis of tumor tissue from 20 breast cancer patients in 2023 [23]. In a recent study, they found that GCSF is one of the tumor-promoting inflammatory cytokines, recruiting a subset of immunosuppressive neutrophils to the brain to drive metastatic growth [20,21].

However, there are limited clinical data elucidating that GCSF use in cancer patients may be associated with distant organ metastases or brain metastases. Until recently, two retrospective studies reported that GCSF is not associated with BM in de novo stage IV breast cancer but is associated with distant organ metastasis in patients with non-small cell lung cancer [24,25]. Therefore, we aimed to examine the potential risks of GCSF use and the incidence of brain metastases using the medical history of stage IV breast cancer patients without BM at diagnosis, since most of them will receive chemotherapy after mastectomy. In addition, we also evaluated the association between GCSF use at different time points and brain metastasis-free survival. These data could be used to develop early plans to prevent or treat the development of brain metastases by tracking the use of GCSF.

## 2. Results

### 2.1. Patient Characteristics

A total of 121 patients were included in the final analysis. Among which, 63 (52%) received GCSF and 58 (47.9%) did not receive GCSF. The clinical characteristics of these GCSF and non-GCSF groups are presented in Table 1. There was no significant difference in the mean age between the two groups (63.5 ± 10.7 years vs. 61.2 ± 12.2 years). Approximately 20.6% and 17.2% patients in GCSF and non-GCSF groups were treated with the epirubicin + cyclophosphamide chemotherapy regimen. Palbociclib was the most common target agent used in this population. The incidence of neutropenia was significantly different in patients who received GCSF versus those who did not (39.7% vs. 67.2, *p* = 0.003).

### 2.2. Prognostic Factors for Brain Metastasis

Thirty of the one hundred and twenty-one stage IV breast cancer patients (28%) had brain metastases during the follow-up period. The incidence of BM in patients using GCSF was significantly higher than that in patients not using GCSF (*p* = 0.011). In univariate and multivariate Cox regression analyses, GCSF treatment was significantly associated with brain metastasis (HR:2.538; 95% confidence interval [CI]: 1.127–5.716), *p* = 0.025 and HR:2.479; 95% CI = 1. 023–6.007; *p* = 0.044). Furthermore, cumulative GCSF doses of ≤4500 μg and density gradients of ≥300 μg/day were significantly associated with BM risk (Table 2).

### 2.3. Impact of GCSF Treatment on the Risk of Brain Metastasis

**Figure 1:**
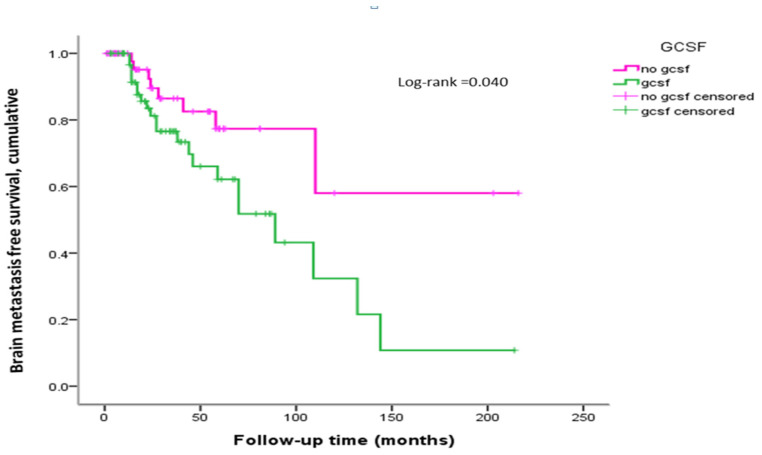
Kaplan–Meier curves of metastasis-free survival (MFS) for advanced stage IV breast cancer patients stratified by No G-CSF and G-CSF.

**Figure 2:**
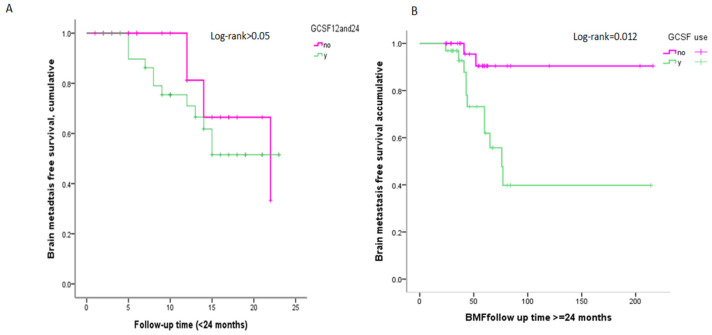
Kaplan–Meier Curve of brain metastasis-free (BMFS) for stage IV breast cancer during follow-up. (**A**) Follow-up stage IV breast cancer < 2 years (24 months). (**B**) Follow-up stage IV breast cancer > 2 years (24 months).

The Kaplan–Meier curve of BMFS in patients with stage IV breast cancer showed that the risk of brain metastasis in the GCSF group was significant compared with the non-GCSF group (Log-rank test < 0.05) (Figure 1). Following GCSF users for ≤2 years or more, GCSF users showed nonsignificant or significant differences in BMFS before being diagnosed with brain metastases compared with non-users (Figure 2).

### 2.4. Subgroup Analysis and Interaction p Test on the Main Corresponding Covariates

Subgroup analysis results showed a similar consistent pattern (Supplementary Figure S1). The interaction *p*-value for most covariates were not significant (interaction *p*-value > 0.05), except for neutropenia and menopause status. In patients who did not develop neutropenia after chemotherapy, GCSF treatment was associated with a significantly increased risk of brain metastases (Risk ratio = 1.84; 95% CI = 1.21, 2.80, interaction *p* = 0.013). In comparison, GCSF treatment reduced the risk of brain metastases in patients who developed neutropenia after chemotherapy (Risk ratio = 0.59; 95% CI = 0.41–0.84, interaction *p* > 0.05). Furthermore, patients with a triple-negative status had poorer BMFS than patients without a triple-negative status (Supplementary Figure S2).

### 2.5. Impact of GCSF Treatment Dose and Density on the Risk of Brain Metastasis

In the univariate Cox regressions looking at the impact of the GCSF cumulative dose or different doses on the risk of brain metastasis, we found that a GCSF cumulative dose < 4500 μg or dose density ≥ 300 (μg/day) significantly increased the risk of brain metastasis (*p* = 0.004, *p* = 0.010) (Table 2). In multivariate Cox regression analysis, we further adjusted for some or all of the covariates to analyze the trend of GCSF increasing brain metastasis (Table 3). For patients treated with GCSF, the incidence of BM in stage IV BC patients without diagnosed BM increased from 13.8% to 34.9% compared with the non-GCSF group, implying a 1.8-fold increase in relative risk in the adjusted I model (HR 2.812; 95% CI 1.248–6.338, *p* < 0.01) and a 2.14-fold increase in relative risk in the adjusted II model (HR 3.144; 95% CI 1.238–7.983, *p* < 0.01).

## 3. Discussion

In this retrospective cohort study, a significant association was found between GCSF use and the risk of brain metastases in this patient population. Although the use of GCSF in combination with cytotoxic chemotherapy to prevent or treat severe neutropenia has shown clear clinical benefits, increasing evidence suggests that the use of GCSF may promote cancer progression and metastasis, producing large quantities in different types of cancers [11]. In addition, Fujii et al. and Wang et al. recently published inconsistent research reports. They concluded that GCSF is not associated with an increased risk of brain metastases in Japanese patients with de novo stage IV breast cancer, but GCSF is associated with an increased risk of distant organ metastases in Chinese patients with non-small cell lung cancer [24,25]. The results of the current study and those of Wang et al. may serve as evidence supporting the effects of GCSF on tumor growth and metastasis [11]. Although GCSF is used in small amounts, it is still considered a hidden harm due to recent biological research on GCSF’s signaling pathways. Considering the overall benefit of GCSF use, delineating the potential role of GCSF in tumor progression could still translate into benefits in patient outcomes [26]. Therefore, we expect more comprehensive large clinical trials to support this issue.

Several studies have shown that GCSF is highly expressed in the TNBC subtype [27,28,29]. The incidence of BM has been reported to be higher (46%) in patients with advanced TNBC than in HR-positive and HER2-positive subtypes of BC [30], but the risk in patients with the HR-negative/HER2-positive subtype is as high as in patients with the TNBC subtype [31,32,33]. At the same time, they found that the poor prognosis of TNBC may be due to the high expression of GCSF, which is significantly related to the estrogen receptor (ER)-negative subtype [27]. This result may support our findings that the effect of GCSF on brain metastases in hormone receptor-positive patients is not significant.

Recently, the specific biology of the ER and HER2 pathways was reported in HER2-positive patients with HR expression. ER signaling is the dominant driver of cell proliferation and survival, including the non-genomic pathway and genomic pathway [34]. The genomic pathway is predominant in tumor cells, inducing ER-related gene transcription and cell proliferation [35]. The nongenomic activation of the HER2 pathway leads to the activation of its downstream pathways, such as mitogen-activated protein kinase (MAPK), protein kinase B (AKT), and PI3K [36]. This hypothesis may also be used to explain the effect of GCSF on the risk of BM in hormonal receptors. High GCSF levels might facilitate cancer cell migration and reduce overall patient survival [27,37]. In a recently published study, they reported that GCSF is an important tumor-related factor that can reduce the level of IRF8 in alveolar macrophages and promote the metastasis of breast cancer with lung metastases. At the same time, they also found that the CD68^hi^IRF8^lo^GCSF^hi^ gene signature in TNBC patients is significant to tumor prognosis [29]. This result may support our finding that patients with a triple-negative status have worse BMFS than patients without a triple-negative status.

Most patients received GCSF for the treatment of chemotherapy-induced neutropenia, according to our health insurance drug regulations. In our study, 99% of patients received GCSF at a dose of 16 μg/kg/day and 5 μg/kg/day for the treatment and prevention of neutropenia. This appears to be consistent with the literature and oncology clinical expertise. In multivariate Cox regression, a cumulative dose of GCSF < 4500 μg and a dose density of ≥300 μg/day showed significant association with brain metastasis. This result may be consistent with those suggested in a recently published study, which indicated that the metastasis-promoting function of GCSF is dose density-dependent rather than completely dose-dependent [25]. Therefore, more precise practice standards are needed to guide the use of GCSF in clinical practice to reduce the risk of brain metastasis. In addition, they also reported that frequent use of GCSF in a short period of time would expose patients to a higher risk of metastasis and worsen prognosis [25]. This hypothesis also prompts us to think deeply about whether the short-term use of high-frequency GCSF should be avoided in clinical practice. Although the benefits of GCSF are clear, they are accompanied by an increased risk of brain metastasis.

The biological mechanism of brain metastasis in breast cancer patients is complex and may be caused by the dysregulation of multiple cellular signaling pathways, such as PI3K, AKT, JAK-STAT3, MAPK-ERK1, NF-κB, Wnt–β-catenin STAT3, p53, TGF-β, EGF, NF-Kβ, and others [32,38]. Cancer cells metastasize to the brain and interact with the local tumor microenvironment to destroy the blood–brain barrier (BBB), leading to brain metastasis; at the same time, in tumor cells, the activation of JAK/STAT3 and PI3K/AKT signaling pathways and the downregulation of phosphatases and tensin homolog (PTEN) develop cell cycle progression, tumor cell proliferation, and, finally, reduced survival [39].

To compare with the biology of GCSF signaling, we hypothesize that STAT3 is one of the signaling transcription factors (transcription factors (STAT 1, 3, and 5)) triggered when GCSF binds to its receptor, which then activates three major signaling pathways: JAK/STAT3, PI3K/AKT, and MAPK/ERK [11]. Under normal circumstances, these three signaling pathways activate NF-κB and C/EBPβ transcription factors and then bind to the regulatory elements of the GCSF promoter to induce neutrophil activation in the bone marrow. However, the aberrant activation of signaling pathways or mutations in the GCSF receptor pathway may affect the myeloid lineage and ultimately be directly associated with malignancy. Therefore, we reasonably speculate that there are similar signaling molecules, such as NF-κB and STAT3, between brain metastases and GCSF transcriptional signaling pathways. This complex biology of GCSF signaling may explain the significantly increased incidence of GCSF-induced brain metastases in our study [11].

In breast cancer, a patient’s tumor mass is frequently infiltrated by neutrophils (tumor-associated neutrophils, TANs). Studies demonstrated that TANs in human BC have detrimental effects and support malignant cell invasion and migration. Increasing evidence suggests that cytokines, including IL-8 and IL-6, can alter the phenotype of neutrophils and thereby produce tumor-promoting effects [40]. A study conducted by Sheng et al. reported that GCSF was found to be a key component of MDA231CS, which extended the lifespan and metastatic ability of TANs. They also showed a positive correlation between TAN and GCSF expression [11]. These results may support our subgroup analysis which found that the interaction *p* value was significant in neutropenic patients treated with GCSF.

This study has several limitations. First, this study was retrospective and therefore may have had some selection bias. Second, this study focused on stage IV breast cancer because early-stage breast cancer rarely develops brain metastases, which may have affected the sample size collected at only one institution. Finally, the small sample size and population differences in the Her2 and TNBC subtypes between the two cohorts may contribute to population heterogeneity. The robustness of the subgroup analysis may offset its limitations. However, further studies using large data sets from Health Insurance Research Databases are needed to confirm the effect of GCSF on brain metastases.

## 4. Materials and Methods

### 4.1. Study Population

Our institutional review board approved this retrospective cohort study (protocol number: TMANH112-REC003). We conducted a retrospective cohort study to identify female patients with stage IV BC from the claims database of southern Taiwan regional teaching hospitals based on the International Classification of Diseases, 9th Revision, Clinical Modification (ICD-9-CM) code (174.9) or the International Classification of Diseases, 10 th Revision, Clinical Modification (ICD-10-CM) code (C50) from January 2014 to 31 December 2022. A total of 1412 female patients were retrieved and separated into GCSF users and non-users linked with GCSF ATC codes (L03AA02). We further identified patients with brain metastases using ICD codes (ICD-9: 198.3; ICD-10: C71.9) after the GCSF prescription date. We then reviewed the electronic medical records (EMRs) of the retrieved cohort, identifying patients with stage IV breast cancer based on the following selection criteria: (1) eligible women were older than 18 years with histologically confirmed stage IV BC; (2) no confirmed diagnoses include brain metastases; and (3) they have been treated with at least one cycle of any type of chemotherapy (with or without GCSF) prescribed by an oncologist according to the guidelines at the Southern Taiwan Regional Teaching Hospital [6,41]. We excluded patients diagnosed with coexisting or previous malignancies. We also excluded patients who developed brain metastases before GCSF administration. The follow-up time was estimated from the date of BM diagnosis, the last follow-up, or the date of loss to follow-up. The last tracking date was 31 December 2022.

### 4.2. Data Collection and Treatment

We reviewed the EMRs to extract patient characteristics, including age, menopausal status at diagnosis, hormone receptor status, HER2 receptor status, smoking status, hormone treatment, HER2 treatment, immunotherapy, brain metastases, myelosuppression, and neutropenia status. Estrogen receptor (ER) and/or progesterone receptor (PR) positivity were defined based on the immunohistochemistry results [42]. HER2 positivity was defined as a HER2/CEP17 ratio in fluorescence in situ hybridization (FISH) of 2.0 and/or an immunohistochemical staining score of 3+ [43]. GCSF was used to treat and prevent chemotherapy-induced myelosuppression or neutropenia.

We divided patients into two groups according to the use or non-use of GCSF as selection criteria. All GCSF users had at least one GCSF prescription. The effect of GCSF exposure dose on the risk of brain metastases in stage IV breast cancer without BM at diagnosis was analyzed. Two subgroups were established based on the cumulative total GCSF dosage (≤4500 μg and ≥4500 μg). The dose density of GCSF, defined as the dose of GCSF administered over a certain length of time, was used to assess the impact of the GCSF treatment density on the outcome because the dose administration is affected by time. The dose density of GCSF therapy was calculated based on the GCSF exposure time in days from the first prescription to the last prescription (in days). We also evaluated the impact of TNBC status on BMFS.

### 4.3. Outcome Assessments

The primary outcome was the incidence of brain metastases. The secondary outcome was brain metastasis-free survival (BMFS). BMFS was defined as the time from the date of diagnosis of stage IV breast cancer to the radiographic evaluation of brain metastases. If the patient was alive, BMFS was censored at the last follow-up date. Associations between covariates were also assessed.

### 4.4. Statistical Analysis

Descriptive statistics were used to summarize the relationships between different categorical variables and to visualize the proportion of cases in subgroups. The association between two categorical variables was assessed using chi-squared and Fisher’s exact test. Univariate Cox regression analysis was used to evaluate GCSF use and individual covariates (menopausal status at diagnosis, hormone receptor status, HER2 receptor status, smoking status, hormone treatment, HER2 treatment, target therapy, brain metastasis, myelosuppression, neutropenia status, cumulative dosage (μg), dosage density (μg/day), and number of chemotherapy) on the incidence of brain metastases. Multivariable Cox regression analysis was also used to test the independent effects of GCSF and covariates on BM, with *p* values < 0.05 in univariate results only. The significance level was assumed to be *p* < 0.05. BMFS used Kaplan–Meier estimation and log-rank tests. All analyses were performed in SPSS Statistics 28.0.1.1.

### 4.5. Subgroup Analysis and Subgroup Interaction p Test

Subgroup interaction analysis is typically performed to assess statistically significant subgroup differences by calculating separately the multiplicative terms of the main categorical covariates in the model. If the interaction *p*-value was significant, we concluded that the effect of the intervention on the outcome differs within subgroups [44]. We also performed subgroup analysis using RevMan 5.4 version to create a forest tree to further confirm whether GCSF was an independent adverse factor for metastasis and was robust to major covariates [25].

## 5. Conclusions

In patients with stage IV breast cancer receiving chemotherapy, we found that GCSF use was associated with a higher risk of brain metastases, and this risk may increase significantly with the increasing GCSF dose density. Current research suggests greater side effects of GCSF use in patients who develop neutropenia during chemotherapy, and more precise criteria are needed to improve compliance with guideline recommendations for the appropriate use of GCSF and to improve patient safety. Since the mortality rate of stage IV breast cancer is high, our findings can serve as additional reference evidence for the use of GCSF in clinical practice. In addition, more clinical studies on the complex mechanisms of GCSF’s role in the tumor microenvironment may be needed in the near future to further confirm the correlation between GCSF and brain metastasis.

## Figures and Tables

**Table 1:** Patient characteristics in this study.

Characteristics	GCSF (N = 63)	No GCSF (N = 58)	*p* Value
Age (mean ± SD)	63.5 ± 10.7	61.2 ± 12.2	0.542
Female	63 (100)	58 (100)	-
Menopausal status, *n* (%)			0.540
<55 years	15 (23.8)	17 (29.3)	
≥55 years	48 (76.2)	41 (70.7)	
Smoking Status, *n* (%)			NA
Former/current	0	0	
Never	63 (100)	58 (100)	
Hormone receptor status, *n* (%)			0.347
Positive	38 (60.32)	40 (68.97)	
Negative	25 (39.68)	18 (31.03)	
HER2 status, *n* (%)			0.715
Positive	30 (47.6)	25 (39.7)	
Negative	33 (52.4)	33 (60.3)	
TNBC, *n* (%)			0.027 *
Positive	12 (19.1)	3 (4.76)	
Negative	51 (80.9)	55 (95.24)	
Hormone treatment, *n* (%)			0.717
Yes	29 (46.0)	29 (50.0)	
No	34 (54.0)	29 (50.0)	
HER2 treatment, *n* (%)			0.106
Yes	22 (34.9)	12 (20.9)	
No	50 (65.1)	45 (79.1)	
Targeted drug therapy, *n* (%)			0.828
Yes	13 (15.2)	13 (20.7)	
No	41(84.8)	46 (79.3)	
Brain metastasis, *n* (%)			0.011 *
Yes	22 (34.9)	8 (13.8)	
No	41 (65.1)	50 (86.2)	
Chemotherapy, *n* (%)			
Epirubicin + Cyclophosphamide	12 (20.6)	11 (17.2)	0.588
Eribulin	12 (17.5)	6 (12.1)	0.138
Taxane/docetaxel	22 (34.9)	22 (37.9)	0.438
Others ^+^	17 (26.9)	19 (32.8)	0.659
Chemotherapy items ≥ 2			<0.0001
<2	17 (27.0)	44 (75.9)	
≥2	46 (73.0)	14 (24.1)	
Myelosuppression, *n* (%)			0.845
Yes	20 (31.7)	17 (29.3)	
No	43 (68.3)	41 (70.7)	
Neutropenia, *n* (%)			0.003 *
Yes	25(39.7)	39 (67.2)	
No	38 (60.3)	19 (32.8)	
Dosage density (μg/day)			<0.0001
0	0 (0)	58 (100)	
<300	40 (62.5)	0 (0)	
≥300	23 (37.5)	0 (0)	
Dosage, cumulated (μg)			
0	0 (0)	58 (100)	<0.0001
≤4500	31 (49.2)	0 (0)	
>4500	32 (50.8)	0 (0)	

Remarks: *n*, number of variables; μg, microgram which is one millionth of a gram or one thousandth of a milligram;GCSF, granulocyte colony-stimulating factor. *p* < 0.05 statistical significance, patient characteristics between two groups. * *p* < 0.05, statistic significant. Others ^+^: Gemcitabine, Navelbine, Carboplatin

**Table 2:** Effects of risk factors on brain metastasis by univariate and multivariate Cox analysis.

		Univariate		Multivariate	
Variables (Risk Factors)	*n* (%)	HR (95%CI)	*p* Value	HR (95%CI)	*p* Value
GCSF use	63 (52.1)	2.538 (1.127–5.716)	0.025 *	2.479(1.023–6.007)	0.044 *
Menopause Status	89 (73.6)	4.062 (1.968–8.384)	0.000 *	3.305 (1.521–7.179)	0.003 *
Hormone treatment	79 (65.3)	2.713 (1.261–5.836)	0.011 *	2.266 (1.025–5.011)	0.043 *
Hormone receptor status	78 (64.5)	1.557 (0.738–3.285)	0.245	--	
HER2 treatment	34 (28.1)	2.800(1.331–5.889)	0.007 *	2.159 (1.013–4.603)	0.046 *
HER2 Status	55 (45.5)	1.138 (0.543–2.384)	0.731	--	--
TNBC	15 (12.4)	0.381 (0.142–1.018)	0.054 *	---	---
Target therapy	26 (21.5)	0.600 (0.254–1.414)	0.243	--	---
Myelosuppression	37 (30.6)	1.392 (0.596–3.251)	0.445	---	---
Neutropenia	46 (38.0)	3.276 (1.530–7.013)	0.002 *	2.418 (1.054–5.548)	0.037 *
Dosage, cumulated (μg)					
0	58 (47.9)	1	1	--	--
≤4500	30 (24.8)	1.896(1.223–2.940)	0.004 *	1.296 (0.449–3.743)	0.632
>4500	33 (27.3)	1.255(0.817–1.929)	0.299	----	---
Dosage density (μg/day)					
0	58 (47.9)	1	1	--	---
<300	40 (33.1)	1.353(0.868–2.107)	0.182	---	---
≥300	24 (19.8)	1.753(1.142–2.691)	0.010 *	1.866 (1.212–2.872)	0.005 *
No. of Chemotherapy					
<2	58(47.9)	0.664 (0.315–1.398)	0.281	---	----
≥2	63 (52.1)	0.890 (0.431–1.842)	0.754	---	---

Remarks: HR: Hazard ratio; CI: Confidence interval; (μg) microgram which is one millionth of a gram or one thousandth of a milligram; GCSF: granulocyte colony-stimulating factor; n (%): number of risk factors/percentage of total patients = 121). HR+ (estrogen receptor or progesterone receptor positive).* statistical significance.

**Table 3:** Trend test for the effect of GCSF treatment dose and density on risk of metastasis.

	Total N	Event n%	Non-Adjusted Model HR (95% CI)	*p* Value	Adjust Model I HR (95%CI)	*p* Value	Adjusted Model II HR (95%CI)	*p* Value
**GCSF**								
No GCSF	58	8 (13.8)	1		1		1	
GCSF	63	22 (34.9)	2.538 (1.127–5.716)	0.025	2.812 (1.248–6.338)	0.013	3.144 (1.24–7.98)	0.016
Dosage, cumulated (μg)								
≤4500	63	31 (49.2)	2.068 (1.263–3.385)	0.004	1.685 (1.09–2.603)	0.019	0.358 (0.120–1.066)	0.065
>4500	58	32 (55.2)	0.255 (0.817–1.929)	0.299	1.101 (0.708–1.711)	0.669	0.507 (0.176–1.460)	0.208
*p* for trend			0.005		0.003		0.027	
Dosage density (μg/day)								
<300	63	40 (63.4)	1.353 (0.868–2.107)	0.182	1.208 (0.771–1.894)	0.409	0.775 (0.217–2.768)	0.695
≥300	58	23 (39.7)	1.753 (1.142–2.691)	0.010	0. 153 (1.004–2.344)	0.048	2.285 (1.224–4.264)	0.009
*p* for trend			0.032		0.033		0.051	

Remarks: HR, Hazard ratio; CI, Confidence interval; μg, microgram which is one millionth of a gram or one thousandth of a milligram; GCSF, granulocyte colony-stimulating factor; Non-adjusted model, adjust for none; Adjusted model I, adjust for age and menopause; Adjusted model II, adjust for age, menopause, hormone status, hormone treatment, HER2 status, HER2 treatment, TNBC, number of chemotherapy, and neutropenia.

## Data Availability

Data are provided within the manuscript or Supplementary Information Files.

## Supplementary Materials

The following supporting information can be downloaded at https://www.mdpi.com/article/10.3390/ijms251910756/s1.
