# Supplementary material for: Association of Granulocyte Colony-Stimulating Factor Treatment with Risk of Brain Metastasis in Advanced Stage Breast Cancer
Source: Int J Mol Sci. 2024 Oct 6;25(19):10756. doi: 10.3390/ijms251910756 (PMC11477282; doi:10.3390/ijms251910756)
Supplement: Supplementary file 1 [file ijms-25-10756-s001.zip › ijms-3211213-supplementary.pdf]

	N	Risk ratio	Risk ratio (95% CI)	Interaction P value
Her2 status (-)	66		0.92 [0.67, 1.27]	0.183
Her2 status +	55		1.10 [0.75, 1.64]	
Her-2 treatment No	87		0.82 [0.66, 1.03]	0.108
Yes	34		1.69 [0.92, 3.09]	
Hormone status (-)	43		1.28 [0.78, 2.09]	0.654
(+)	78		0.87 [0.67, 1.14]	
Hormone treatment No	63		1.08 [0.77, 1.52]	0.123
Yes	58		0.92 [0.64, 1.33]	
Menopause <55years	32		0.81 [0.45, 1.47]	0.962
≥55 years	89		1.08 [0.87, 1.34]	
Myelosuppression (-)	84		0.97 [0.76, 1.22]	0.656
Myelosuppression (+)	37		1.08 [0.63, 1.86]	
Neutropenia No	57		1.84 [1.21, 2.80]	0.013*
Neutropenia Yes	64		0.59 [0.41, 0.84]	
Chemotherapy <2	61		0.36 [0.23, 0.55]	0.898
Chemotherapy >=2	60		3.02 [1.87, 4.89]	
TNBC No	106		0.94 [0.80, 1.10]	0.544
Yes	15		3.05 [0.91, 10.20]	

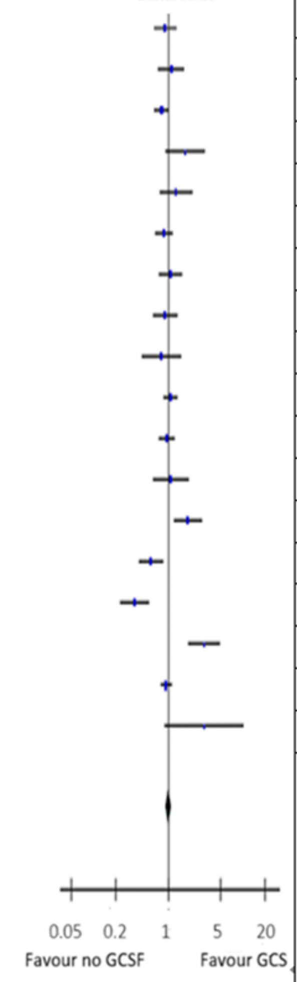

Figure S1. Subgroup analysis and subgroup interaction p test on effect of G-CSF on brain metastasis in aBC patients (reference 29) N=total patient number

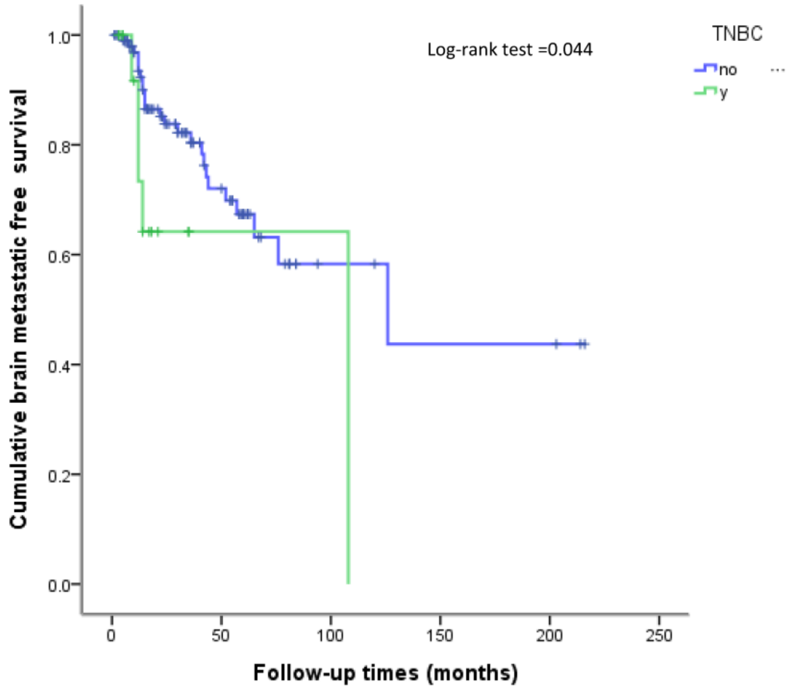

Figure S2. Cumulative brain metastatic free survival in patients with TNBC vs non TNBC status
